# *Wolbachia* Inter-Strain Competition and Inhibition of Expression of Cytoplasmic Incompatibility in Mosquito

**DOI:** 10.3389/fmicb.2020.01638

**Published:** 2020-07-10

**Authors:** Xiao Liang, Julian Liu, Guowu Bian, Zhiyong Xi

**Affiliations:** ^1^Department of Microbiology and Molecular Genetics, Michigan State University, East Lansing, MI, United States; ^2^Guangzhou Wolbaki Biotech Co., Ltd., Guangzhou, China

**Keywords:** *Wolbachia*, dengue, cytoplasmic incompatibility, *Aedes albopictus*, transinfection, inter-strain competition

## Abstract

Successful field trials have been reported as part of the effort to develop the maternally transmitted endosymbiontic bacteria *Wolbachia* as an intervention agent for controlling mosquito vectors and their transmitted diseases. In order to further improve this novel intervention, artificially transinfected mosquitoes must be optimized to display maximum pathogen blocking, the desired cytoplasmic incompatibility (CI) pattern, and the lowest possible fitness cost. Achieving such optimization, however, requires a better understanding of the interactions between the host and various *Wolbabachia* strains and their combinations. Here, we transferred the *Wolbachia w*Mel strain by embryonic microinjection into *Aedes albopictus*, resulting in the successful establishment of a transinfected line, HM (*w*AlbA*w*AlbB*w*Mel), with a triple-strain infection comprising *w*Mel, *w*AlbA, and *w*AlbB. Surprisingly, no CI was induced when the triply infected males were crossed with the wild-type GUA females or with another triply infected HC females carrying *w*Pip, *w*AlbA, and *w*AlbB, but specific removal of *w*AlbA from the HM (*w*AlbA*w*AlbB*w*Mel) line resulted in the expression of CI after crosses with lines infected by either one, two, or three strains of *Wolbachia*. The transinfected line showed perfect maternal transmission of the triple infection, with fluctuating egg hatch rates that improved to normal levels after repeated outcrosses with GUA line. Strain-specific qPCR assays showed that *w*Mel and *w*AlbB were present at the highest densities in the ovaries and midguts, respectively, of the HM (*w*AlbA*w*AlbB*w*Mel) mosquitoes. These finding suggest that introducing a novel strain of *Wolbachia* into a *Wolbachia*-infected host may result in complicated interactions between *Wolbachia* and the host and between the various *Wolbachia* strains, with competition likely to occur between strains in the same supergroup.

## Introduction

A rapid increase in the number of arbovirus diseases transmitted by mosquitoes, such as dengue and Zika, in recent decades has underscored the urgency in developing effective intervention strategies (Velayudhan, 2012; Katzelnick et al., 2017). The insufficiency of traditional control approaches, including vaccines, drugs, and chemical insecticides, has led to significant efforts to develop novel vector control methods to combat disease transmission. Rather than using chemical insecticides to directly kill the vector, an approach that is being challenged by the rapid development of insecticide resistance and the negative impacts on both the environment and non-target insect species, these new tools have focused on modifying the mosquito population in a species-specific manner, with the goal of either reducing the mosquito’s ability to host a pathogen or suppressing (or even eliminating) the mosquito population to break the viral transmission between vector and host (Achee et al., 2015). Among these strategies, *Wolbachia*-based interventions have recently shown encouraging results in field trials, successfully demonstrating either reduced dengue transmission through *Wobachia*-induced viral inhibition in the mosquitoes or the elimination of the *Aedes* vector populations through *Wobachia*-induced incompatible mating (Hoffmann et al., 2011; Mains et al., 2016; Nazni et al., 2019; Zheng et al., 2019; Crawford et al., 2020).

Estimated to infect >65% of all insect species, *Wolbachia* are maternally transmitted endosymbiotic bacteria belong to the order Rickettsiales and family Anaplasmataceae (Werren et al., 2008). Designated based on their naturally associated host species and divided into eight supergroups, different *Wolbachia* strains can interact with their hosts in their own manner, with phenotypes determined by the genetic background of both *Wolbachia* and the host, as well as the environment (McGraw et al., 2002; Werren et al., 2008; Ross et al., 2017). In the mosquito and many other insects, *Wolbachia* causes a reproduction alteration known as cytoplasmic incompatibility (CI), in which early embryonic death occurs when the *Wolbachia*-infected male mates with an uninfected female or a female carrying a different strain of *Wolbachia*. The CI can be rescued, resulting in compatible mating, if the *Wolbachia* strain carried by the male is also present in the female. Recent studies have shown that two CI determination genes, *cifA* and *cifB*, in *Wolbachia* modify the sperm development to induce CI, but only *cifA* mediates CI rescue in females (or eggs; LePage et al., 2017; Shropshire et al., 2018; Beckmann et al., 2019; Chen et al., 2019). However, it is still unknown how these CI factors interact with their host targets and how the CI determination factors of different *Wolbachia* strains interact with each other to induce CI expression in a host with a *Wolbachia* superinfection.

Since the ability to generate novel *Wolbachia* symbiosis (transinfection) in mosquitoes was first developed through embryonic microinjection (Xi et al., 2005a, b, 2006), a number of transinfected mosquito lines carrying different *Wolbachia* strains have been established and characterized, with the goal of using them for disease control (Xi et al., 2005b, 2006; McMeniman et al., 2009; Walker et al., 2011; Blagrove et al., 2012; Joubert et al., 2016; Ant et al., 2018; Ross et al., 2019). Many of these transinfected mosquito lines show different levels of resistance to dengue, Zika, and Chikungunya viruses, with the strength of the viral inhibition being associated with the density of *Wolbachia* in somatic tissues such as the midgut and salivary glands, where the viruses reside, migrate, and replicate. Whereas transinfected lines with each of three *Wolbachia* strains – *w*Mel, *w*AlbB, and *w*Pip – have been well characterized and successfully tested in field trials (Hoffmann et al., 2011; Nazni et al., 2019; Zheng et al., 2019), significant interest remains in developing improved transinfected lines with maximal viral blocking and optimal fitness under field conditions in order to reach the highest efficiency in disease control or to be able to replace the released lines if viruses develop resistance to the released strains in the future (Ross et al., 2019).

Naturally carrying two *Wolbachia* strains, *w*AlbA and *w*AlbB, *Aedes albopictus* is the world’s most invasive mosquito vector and an epidemiologically important vector for many arboviruses. As the density of these two native *Wolbachia* is too low to induce viral inhibiton in *Ae. albopictus* (Lu et al., 2012), efforts have been made to introduce novel strains into this mosquito species to develop transinfected lines that are both incompatible with the wild-type line and resistant to viruses (Blagrove et al., 2013; Zheng et al., 2019). Experiments are often designed by either directly adding a novel strain to *Ae. albopictus* to generate a superinfection (Fu et al., 2010; Suh et al., 2016; Ant and Sinkins, 2018; Zheng et al., 2019) or replacing the native *Wolbachia* with a novel strain, by removing the native *Wolbachia* with an antibiotic and then introducing the novel strain (Xi et al., 2006; Blagrove et al., 2012). The first approach results in a triple infection to induce an unidirectional CI with wild-type mosquitoes (Fu et al., 2010; Zheng et al., 2019), with the advantage that *Wolbachia* invade and spread into the population more effectively than does the second (replacement) approach, which often induces a bi-directional CI (Xi et al., 2006; Blagrove et al., 2012). However, for a host with a triple-strain infection, the outcome of the transinfection is difficult to predict, given the complicated interactions between the various *Wolbachia* strains and between *Wolbachia* and the host (Suh et al., 2016; Ant and Sinkins, 2018). When a tripe-strain infection comprising *w*Mel, *w*AlbA, and *w*AlbB was previously established in *Ae. albopictus*, very low egg hatch rates were observed in both the self-cross of the transinfected line and the compatible cross of the transinfected females with wild-type males (Ant and Sinkins, 2018), suggesting that the ability of this *Wolbachia* triple-strain infection to recue CI modification was compromised due to unknown inter-strain interactions.

We previously developed the transinfected *Ae. albopictus* line HC, featuring another triple infection with *w*Pip, *w*AlbA, and *w*AlbB (Zheng et al., 2019). The HC line induces complete unidirectional CI in crosses with the wild-type line, with intact ability of HC females to rescue CI when mated with either wild-type or HC males (Zheng et al., 2019). In the present study, we have introduced *w*Mel into *Ae. albopictus* and generated the transinfected line, HM (*w*AlbA*w*AlbB*w*Mel), infected with *w*Mel, *w*AlbA, and *w*AlbB. The transinfected line show complete efficiency in maternal transmission of the triple infection, with *w*Mel showing the highest density in ovaries. Multiple crosses showed that the ability of *w*Mel to induce CI was blocked by *w*AlbA in the HM (*w*AlbA*w*AlbB*w*Mel) line and that double infection with *w*Mel and *w*AlbB induced a high level of CI in crosses with the lines infected with either a single, double, or triple infection.

## Materials and Methods

### Mosquito Lines and Maintenance

Two wild-type *Ae. albopictus* lines, HOU (Xi et al., 2005a) and GUA (Zheng et al., 2019), carrying a native superinfection with *w*AlbA and *w*AlbB were used in this study. Two transinfected *Ae. albopictus* lines, HB and HC, carrying a single *w*AlbB infection and a triple infection with *w*Mel, *w*AlbA, and *w*AlbB, respectively, had been generated previously (Xi et al., 2005a; Zheng et al., 2019) and were used in the CI crosses. The transinfected *Aedes aegypti* MGYP2 line (Walker et al., 2011), carrying *w*Mel, was used as a donor to generate the HM (*w*AlbA*w*AlbB*w*Mel) line.

All the mosquito lines were maintained on a 10% sugar solution at 27 ± 1°C and 80 ± 10% relative humidity, with a 12:12 h light:dark photoperiod, according to standard rearing procedures. For routine colony maintenance and experimental studies, female mosquitoes were provided with either human (for the MGYP2 line) or sheep (for the other lines) blood at day-7 post-eclosion, and eggs were collected 2 days post-blood meal.

### Transinfection to Generate the HM (*w*AlbA*w*AlbB*w*Mel) Line

The HM (*w*AlbA*w*AlbB*w*Mel) line was generated by transfer of *w*Mel from *Ae. aegypti* MGYP2 to *Ae. albopictus* HOU using embryonic microinjection according to the approach described previously (Xi et al., 2005a, b). In brief, cytoplasm from donor embryos was transferred into the posterior of 60–90-min-old recipient embryos using an IM300 microinjector (Narishige Scientific). After injection, the embryos were incubated at 85% relative humidity and 27°C for 1 h, then transferred to wet filter paper. Embryos were allowed to mature for 5–7 days before hatching. Females (G0) developing from the surviving embryos were isolated and mated with HOU males. After blood-feeding and oviposition, G0 females were tested for *w*Mel infection by PCR using strain-specific primers as described below. G1 females were again crossed with HOU males, blood-fed, isolated, and allowed to oviposit. The offspring from *w*Mel-positive G1 were selected for the next screen, and this process was repeated until the *w*Mel maternal transmission rate reached 100%. Diagnosis of *Wolbachia w*AlbA and *w*AlbB was also performed to ensure that the transinfected line carried the triple infection.

### PCR Assays of *Wolbachia* Infection

Primers were designed for strain-specific diagnosis of four different strains on the basis of the sequence of the gene encoding the *Wolbachia* surface protein *wsp*. The primers for *w*AlbA were: forward 5′-GTGTTGGTGCAGCGTATGTC-3′; reverse 5′-GCACCAGTAGTTTCGCTATC-3′. The primers for *w*AlbB were: forward 5′-ACGTTGGTGGTGCAACATTTG-3′; reverse 5′-TAACGAGCACCAGCATAAAGC-3′. The primers for *w*Mel were: forward 5′-CCTTTGGAACCCGCTGTGAATG-3′; reverse 5′-GCCTGCATCAGCAGCCTGTC-3′. The primers for *w*Pip were: forward 5′-TATTTCCCACTATATCCCTTC-3′; reverse 5′-GGATTTGACCTTTCCGGC-3′. The primers given below for mosquito rps6 have been reported previously (Molina-Cruz et al., 2005): forward 5′-CGTCGTCAGGAACGTATTCG-3′; and reverse 5′-TCTTGGCAGCCTTGACAGC-3′. Standard curves were generated for each of the genes listed above to convert the Ct value from quantitative PCR (qPCR) to the copy number of target sequences.

Genomic DNA was extracted from the samples using a Thermo Scientific Phire Animal Tissue Direct PCR Kit (F-140WH). Samples were pre-treated in 20 μl of dilution buffer with 0.5 μl DNARelease Additive. The reaction mixture contained 10 μl 2X Phire Animal Tissue PCR Buffer, 0.4 μl Phire Hot Start II DNA Polymerase, 0.2 μl of both the forward and reverses primer, and 7.2 μl dsH_2_O. The regular PCR conditions were: initial denaturation at 98°C for 6 min, followed by 40 cycles of 5 s at 98°C, 5 s at 56°C, and 45 s at 72°C. qPCR was performed using a QuantiTect SYBR Green PCR Kit (Qiagen) and ABI Detection System ABI Prism 7000 (Applied Biosystems, Foster City, CA, United States). Samples were homogenized in 100 μl 1× STE buffer and incubated with 4 μl of roteinase K at 55°C for 1 h, followed by 97°C for 5 min.

### Tetracycline Treatment of the HM (*w*AlbA*w*AlbB*w*Mel) Line to Generate the HM2 (*w*AlbB*w*Mel) Line With a Double Infection of *w*Mel and *w*AlbB

Once the HM (*w*AlbA*w*AlbB*w*Mel) mosquitoes had emerged as adults (day 0), they were provided with 0.5 mg/ml tetracycline HCl in a 10% sugar solution. This solution was replaced with a 10% sugar solution from day 3 or 4, and a blood meal was provided on day 7. Two days after the blood feeding, the mosquitoes were provided with oviposition cups containing wet filter paper. These treatments were repeated for four generations. At G3, after blood-feeding, the females were isolated for oviposition. After their eggs collected, individual isofemales were sacrificed to extract genomic DNA, and a PCR assay was used to identify each of the three *Wolbachia* strains. Only the eggs from females showing a double infection with *w*Mel and *w*AlbB were allowed to hatch to establish the line. The isofemale selection described above was repeated at G5 to ensure the removal of *w*AlbA, and the resulting HM2 (*w*AlbB*w*Mel) line carried only the double infection with *w*Mel and *w*AlbB.

### Experimental Crosses to Determine CI

Cytoplasmic incompatibility assays were conducted as previously described (Xi et al., 2005a, b). A total of 10 virgin males were mated with 10 virgin females in five replicate cages for each cross. A blood meal was provided to the females at day 7 post-eclosion. Two days after the blood meal, eggs were collected using oviposition cups containing wet filter paper, which was subsequently desiccated for 7 days at 27°C and 80% relative humidity. The eggs were counted and then hatched in water containing 6% (m/v) bovine liver powder. Larvae were counted at the L2-L3 stage to record the hatch rate.

### Statistical Analysis

All data were statistically analyzed by GraphPad Prism 5.0 software. ANOVA and Tukey’s multiple comparisons test were used to compare egg hatching in CI cross experiment and density of each *Wolbachia* strain in mosquito salivary glands, midguts, and ovaries.

## Results

### Generation of the *Ae. albopictus* Transinfected Line With a Triple *Wolbachia* Infection: *w*Mel, *w*AlbA, and *w*AlbB

**FIGURE 1:**
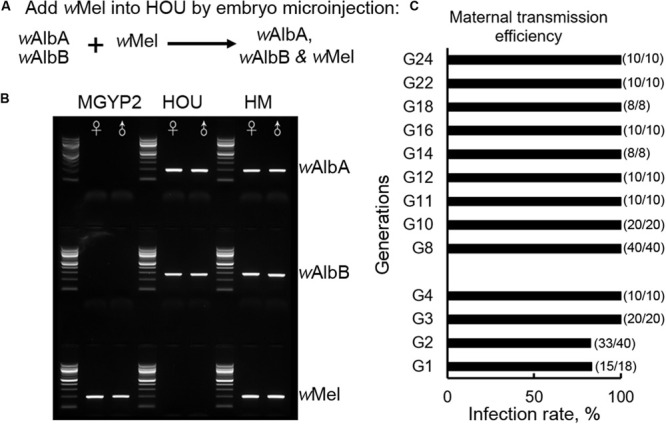
Establishment of the transinfected *Ae. albopictus* HM (*w*AlbA*w*AlbB*w*Mel) line with a triple infection. **(A)** Schematic diagram of the experimental design to establish the HM (*w*AlbA*w*AlbB*w*Mel) line with a *Wolbachia* triple-strain infection. **(B)** Representative results of the strain-specific amplification of the three *Wolbachia* strains, *w*Mel, *w*AlbA, and *w*AlbB, in a PCR assay. MGYP2, the transinfected *Aedes aegypti* line carrying *w*Mel, serving as a donor during the embryonic microinjection experiment in this study. HOU, *Ae. albopictus* HOU line carrying *w*AlbA and *w*AlbB, serving as the recipient. HM, transinfected *Ae. albopictus* line carrying the triple infection *w*Mel, *w*AlbA, and *w*AlbB. **(C)** Maternal transmission efficiency was monitored by randomly selecting individuals from each generation, as indicated, and diagnosis of *Wolbachia* infection by PCR using strain-specific primers. The infection rate was calculated as the percentage of positive individuals in the tested samples at the designated generation.

The ability of a single *w*Mel infection to inhibit arbovirus transmission in both *Ae. aegypti* and *Ae. albopictus* (Walker et al., 2011; Blagrove et al., 2012) motivated us to test whether a triple infection with *w*Mel, *w*AlbA, and *w*AlbB could be established in *Ae. albopictus* to produce enhanced viral blocking effects for disease control, and whether there was competition among the various *Wolbachia* strains that might affect the nature of the symbiosis between *Wolbachia* and its mosquito host. The cytoplasm of *w*Mel-infected *Ae. aegypti* (MGYP2) embryos (Walker et al., 2011) was transferred by microinjection into embryos of the *Ae. albopictus* HOU line with a native superinfection of *w*AlbA and *w*AlbB (Figure 1A). The virgin females (G0) developed from embryos surviving the microinjection were outcrossed with HOU males to produce offspring (G1). A total of 18 G1 isofemales were outcrossed with HOU males. After their eggs (G2) were collected, PCR assay was used to diagnose the *Wolbachia* strain profile in these females, with 15 of 18 isofemales (83%) being seen to carry the triple *Wolbachia* infection (Figures 1B,C); the offspring of the females without a triple infection were discarded. Among the G2 offspring of these triply infected mothers, 18 of 20 (90%) males and 15 of 20 (75%) females maintained a triple *Wolbachia* infection. Without further screening, the offspring from the triply infected G2 females were then pooled together to establish a new transinfected line, hereafter referred to as HM (*w*AlbA*w*AlbB*w*Mel). At G3 and G4, we randomly selected 20 and 10 individuals, respectively, for PCR assay. All of the tested mosquitoes carried a triple infection, indicating a 100% maternal transmission efficiency. Subsequently, the infection status of the HM (*w*AlbA*w*AlbB*w*Mel) line was monitored every other generation from G8 to G24, and all the tested samples (*n* = 126) were positve, confirming the stability of the triple infection in the HM (*w*AlbA*w*AlbB*w*Mel) line (Figure 1C). These results suggest that *w*Mel can coexist with *w*AlbA and *w*AlbB to exhibit symbiosis within *Ae. albopictus*.

### Failure of the HM (*w*AlbA*w*AlbB*w*Mel) Line to Induce CI When Crossed With Wild-Type or Transinfected Lines

The ability to induce CI is a key feature that is required in order to develop *Wolbachia*-based strategies for mosquito-borne disease control. We therefore set up a series of reciprocal crosses among the HM (*w*AlbA*w*AlbB*w*Mel), GUA, and HC lines to measure the relative strength of the *w*Mel-mediated CI expression. All compatible crosses yielded egg hatch rates ranging from 51 to 56% (Table 1). Unexpectedly, two presumably incompatible crosses, matings between HM (*w*AlbA*w*AlbB*w*Mel) males and either GUA or HC females, resulted in high egg hatch rates (46.7 and 43.2%, respectively), indicating compatible mating between them. In contrast, consistent with the ability of HC males to induce a strong CI when crossed with GUA females (Zheng et al., 2019), near-complete CI was observed in the crosses between HC males and HM (*w*AlbA*w*AlbB*w*Mel) females (Table 1). These results indicate that the ability of *w*Mel to induce CI, as observed previously (Walker et al., 2011; Blagrove et al., 2012), is blocked in the HM (*w*AlbA*w*AlbB*w*Mel) line when it co-exists with *w*AlbA and *w*AlbB.

**TABLE 1:** Results of CI crosses among the HM (*w*AlbA*w*AlbB*w*Mel), GUA, and HC lines.

**Expected CI type**	**Cross (♀ × ♂)**	**Infection type**		Percent egg hatch*
		**Female**	**Male**	
Incompatible	HC × HM	*w*AlbA,*w*AlbB, *w*Pip	*w*AlbA,*w*AlbB, *w*Mel	43.2 ± 11.6 a
	HM × HC	*w*AlbA,*w*AlbB, *w*Mel	*w*AlbA,*w*AlbB, *w*Pip	0.01 ± 0.04 b
	GUA × HM	*w*AlbA,*w*AlbB	*w*AlbA,*w*AlbB, *w*Mel	46.7 ± 7.8 c
Compatible	HM × GUA	*w*AlbA,*w*AlbB, *w*Mel	*w*AlbA,*w*AlbB	55.5 ± 17.8 c
	HM × HM	*w*AlbA,*w*AlbB, *w*Mel	*w*AlbA,*w*AlbB, *w*Mel	55.1 ± 9.0 c
	HC × HC	*w*AlbA,*w*AlbB, *w*Pip	*w*AlbA,*w*AlbB, *w*Pip	51.3 ± 11.7 c

* Expressed as the mean for 15 replicates/cross type ± standard deviation. Different letters following the data indicate significant differences (*P* < 0.001) by ANOVA-Tukey’s multiple comparison test.

### CI Induction by *w*Mel After Removal of *w*AlbA From the HM (*w*AlbA*w*AlbB*w*Mel) Line in *Ae. albopictus*

**FIGURE 2:**
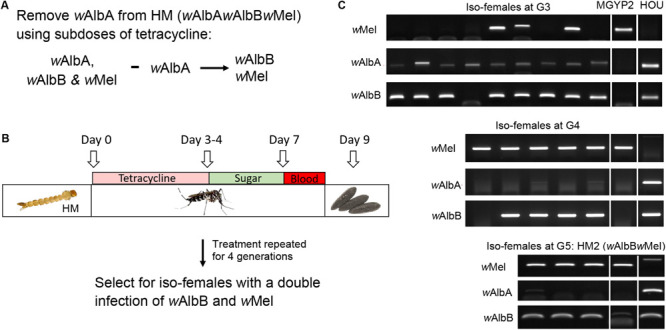
Establishment of the HM2 (*w*AlbB*w*Mel) line carrying the double infection with *w*AlbB and *w*Mel. **(A,B)** Schematic diagram of the experimental approach to remove *w*AlbA from the triply infected HM (*w*AlbA*w*AlbB*w*Mel) line using subdoses of tetracycline. **(C)** Representative results from the PCR screening for isofemales carrying a double infection with *w*AlbB and *w*Mel. *w*AlbA was specifically removed after treatment for four consecutive generations.

In order to understand whether the ability of *w*Mel to induce CI in the HM (*w*AlbA*w*AlbB*w*Mel) line is being blocked by the other two native *Wolbachia* strains, we treated the HM (*w*AlbA*w*AlbB*w*Mel) line with a subdose of tetracycline for four generations and monitored the infection profile by strain-specific PCR from G3 to G5 after tetracycline treatment (Figures 2A–C). This treatment resulted in the specific removal of *w*AlbA from the HM (*w*AlbA*w*AlbB*w*Mel) line and establishment of the HM2 (*w*AlbB*w*Mel) line, with a double infection of *w*Mel and *w*AlbB (Figure 2C). CI crosses were then performed using HM2 (*w*AlbB*w*Mel), GUA, HC, and an *Ae. albopictus* HB line with a single *w*AlbB infection. Strikingly, we observed a strong, although not complete, CI when HM2 (*w*AlbB*w*Mel) males were crossed with GUA, HC, or HB females (Table 2). As expected, HM2 (*w*AlbB*w*Mel) induced bi-directional CI when crossed with the GUA and HC lines, but uni-directional CI when crossed with the HB line. Among all of these incompatible crosses, HC males induced the highest level of CI, with 100% embryonic death. These results indicate that *w*AlbA may block the expression of CI by *w*Mel in the HM (*w*AlbA*w*AlbB*w*Mel) line.

**TABLE 2:** Results of CI crosses among the HM2 (*w*AlbB*w*Mel), GUA, HB, and HC lines.

**Expected CI type**	**Cross (♀ × ♂)**	**Infection types**		Percent egg hatch*
		**Female**	**Male**	
Incompatible	HM2 × HC	*w*AlbB, *w*Mel	*w*AlbA, *w*AlbB, *w*Pip	0 ± 0 a
	HC × HM2	*w*AlbA,*w*AlbB *w*Pip	*w*AlbB, *w*Mel	9.0 ± 7.8 b
	GUA × HM2	*w*AlbA, *w*AlbB	*w*AlbB, *w*Mel	14.6 ± 8.3 b
	HM2 × GUA	*w*AlbB, *w*Mel	*w*AlbA, *w*AlbB	9.3 ± 4.2 b
	HB × HM2	*w*AlbB	*w*AlbB, *w*Mel	14.6 ± 6.1 b
Compatible	HM2 × HB	*w*AlbB, *w*Mel	*w*AlbB	79.6 ± 8.2 c
	HM2 × HM2	*w*AlbB, *w*Mel	*w*AlbB, *w*Mel	64.8 ± 17.7 d
	HB × HB	*w*AlbB	*w*AlbB	83.7 ± 6.9 c
	HC × HC	*w*AlbA, *w*AlbB, *w*Pip	*w*AlbA,*w*AlbB *w*Pip	80.8 ± 7.8 c
	GUA × GUA	*w*AlbA,*w*AlbB	*w*AlbA,*w*AlbB	85.6 ± 4.8 c

* Expressed as the mean for 15 replicates/cross type ± standard deviation. Different letters following the data indicate significant differences (*P* < 0.001) by ANOVA-Tukey’s multiple comparison test.

### Introduction of a New Host Genetic Background Into the HM (*w*AlbA*w*AlbB*w*Mel) Line to Increase Its Fitness

**FIGURE 3:**
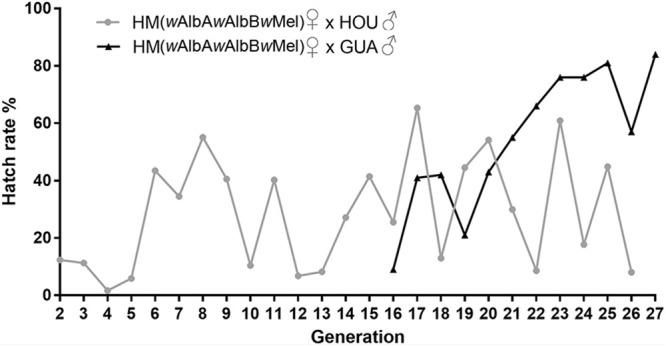
Egg hatch rate of HM (*w*AlbA*w*AlbB*w*Mel) females outcrossed with wild-type males from G2 to G27. Egg hatch was calculated as the percentage of eggs hatched divided by the total number of eggs (see Supplementary Table 1). Outcrosses are indicated as female × male. HM (*w*AlbA*w*AlbB*w*Mel), the transinfected *Ae. albopictus* line carrying the triple infection with *w*Mel, *w*AlbA, and *w*AlbB. HOU and GUA, two wild-type *Ae. albopictus* lines carrying *w*AlbA and *w*AlbB.

The newly established HM (*w*AlbA*w*AlbB*w*Mel) line suffered from a strong fitness cost associated with the triple-strain infection, with an extremely low egg hatch rate ranging from 1 to 12% between G2 and G5 (Figure 3). Therefore, we outcrossed HM (*w*AlbA*w*AlbB*w*Mel) females with HOU males to remove the potential inbreeding effect, which has been observed to cause a low egg hatch rate in previous transinfected lines (Xi et al., 2005a, 2006). The egg hatch rate increased to 60% at G6, then dropped to 12 and 6% at G10 and G12, respectively (Figure 3). From G13 to G27, the egg hatch rate continued fluctuating and varied from 8 to 65%, indicating that the low egg hatch rate may not be only caused by inbreeding; the maladaptation of the novel triple-strain infection to the HOU genetic background may also have contributed to this fitness cost. Thus, at G16, we started to outcross the HM (*w*AlbA*w*AlbB*w*Mel) females with males of the GUA strain, a wild-type *Ae. albopictus* recently collected from the field in Guangzhou, China (Zheng et al., 2019). A steady increase in the egg hatch rate of the outcrossed HM (*w*AlbA*w*AlbB*w*Mel) line was then observed, from 21% at G19 to 84% at G27 (Figure 3), the higher level being similar to that in the GUA strain. Thus, it appears that the GUA genetic background is able to overcome the triple infection-associated decrease in egg hatch rate in the HM (*w*AlbA*w*AlbB*w*Mel) line.

### *w*Mel Distribution in Both the Somatic and Germline Tissues in the HM (*w*AlbA*w*AlbB*w*Mel) Line

**FIGURE 4:**
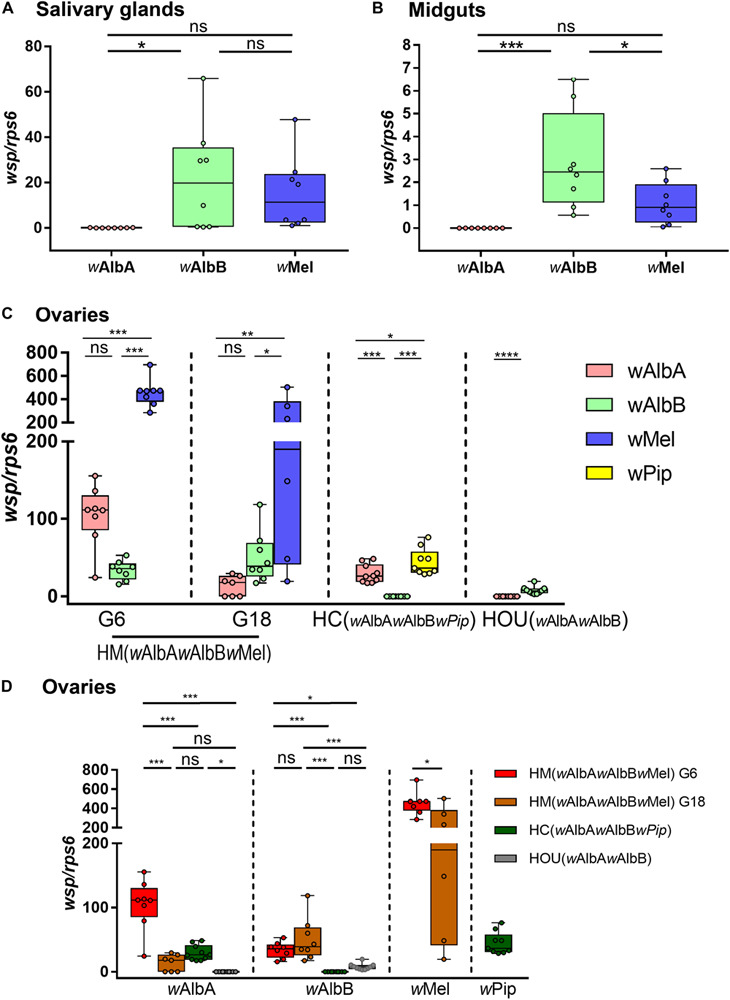
The densities of various *Wolbachia* strains in the salivary glands, midguts, and ovaries of HM (*w*AlbA*w*AlbB*w*Mel) mosquitoes. The densities of *w*Mel, *w*AlbA, and *w*AlbB in salivary glands **(A)** and midguts **(B)** of HM (*w*AlbA*w*AlbB*w*Mel) mosquito were measured by quantitative PCR (qPCR). The densities of the three *Wolbachia* strains in the ovaries of HM (*w*AlbA*w*AlbB*w*Mel) mosquitoes from two generations, G6 and G18, were compared within **(C)** and across **(D)** mosquito lines. HC (*w*AlbA*w*AlbB*w*Pip), the transinfected *Ae. albopictus* line with *w*AlbA, *w*AlbB, and *w*Pip infections. HOU (*w*AlbA*w*AlbB), the wild-type *Ae. albopictus* line with *w*AlbA and *w*AlbB infections. The copy number of the *Wolbachia wsp* gene was normalized by the mosquito *rps6* gene (see Supplementary Table 2). The center of a box plot shows the median of 6–10 replicates, edges show upper and lower quartiles, and bars indicate maximum and minimum values. Dots show values from individual biological replicates. ****P < 0.0001; ***P < 0.001; **P < 0.01; *P < 0.05; ns, not significant; ANOVA and Tukey’s multiple comparisons test.

*Wolbachia* tissue tropism is an important determining factor underlying its viral blocking effect and maternal transmission. We first compared the densities of the three *Wolbachia* strains, *w*Mel, *w*AlbA, and *w*AlbB, in somatic tissues (salivary glands and midgut) and germline tissues (ovaries) of HM (*w*AlbA*w*AlbB*w*Mel) mosquitoes by qPCR. In the salivary glands at G6, the density of *w*AlbB was significantly higher than that of *w*AlbA, but there was no significant difference in density between *w*Mel and *w*AlbA or between *w*Mel and *w*AlbB (Figure 4A). In the midgut, a higher density of *w*AlbB than either *w*Mel or *w*AlbA was observed, whereas the densities of *w*Mel and *w*AlbA did not differ significantly (Figure 4B). These results indicates that *w*AlbB is dominant in the somatic tissue of HM (*w*AlbA*w*AlbB*w*Mel) mosquitoes. In contrast, a higher density of *w*Mel than of *w*AlbA or *w*AlbB is apparent in HM (*w*AlbA*w*AlbB*w*Mel) ovaries (Figure 4C). This distribution pattern was consistently maintained at G6 and G18 despite some degree of fluctuation.

To better understand the strain-specifc interactions in transinfected mosquitoes with triple infections, we also compared the densities of *w*Pip, *w*AlbA, and *w*AlbB in HC ovaries and observed a different order of *Wolbachia* density: *w*Pip > *w*AlbA > *w*AlbB (Figure 4C). Consistent with previous observations (Lu et al., 2012), *w*AlbB was present at a higher level than was *w*AlbA in the ovaries of HOU mosquitoes, from which both the HC and HM (*w*AlbA*w*AlbB*w*Mel) lines were originally derived (Figure 4C). We further compared the density of the same *Wolbachia* strain in ovaries across various mosquito lines to examine the impact of the host’s genetic background on infection levels. *w*AlbA showed its highest level of infection in HM (*w*AlbA*w*AlbB*w*Mel) ovaries at G6 but decreased by 7.8-fold at G18, when it reached a level closer to that in HOU ovaries. The density of *w*AlbB was stable in HM (*w*AlbA*w*AlbB*w*Mel) ovaries from G6 to G18 and was consistently maintained at a level significantly higher than that in both the HOU and HC lines (Figure 4D). The density of *w*Mel decreased by 47% in HM (*w*AlbA*w*AlbB*w*Mel) ovaries from G6 to G18 (Figure 4D) but was still much higher than that of the other *Wolbachia* strains. Interestingly, as compared to HOU ovaries, *w*AlbA was 1,022-fold higher in HM (*w*AlbA*w*AlbB*w*Mel) ovaries at G6, and *w*AlbB was 1,411-fold lower in HC ovaries (Figure 4D). Taken together, these results indicate that *Wolbachia* density is regulated in triply infected *Ae. albopictus* in a strain-, host-, and temporally specific manner.

## Discussion

We have demonstrated the successful establishment of a novel triple *Wolbachia* infection with *w*Mel, *w*AlbA, and *w*AlbB in the *Ae. albopictus* HM (*w*AlbA*w*AlbB*w*Mel) line, with 100% maternal transmission efficiency. Experimental crosses showed that CI is not induced when HM (*w*AlbA*w*AlbB*w*Mel) males mate with either GUA or HC females, but removal of *w*AlbA from the HM (*w*AlbA*w*AlbB*w*Mel) mosquitoes results in CI when these mosquitoes are crossed with three *Ae. albopictus* lines carrying either a single- (HB), double- (GUA), or triple- (HC) strain infection. Despite a severe reduction in the egg hatch rate associated with the triple infection, the rate was returned to normal levels by outcrossing with the wild-type GUA line, but not the HOU line. Among three different *Wolbachia* strains, *w*Mel and *w*AlbB were highest in density in the ovaries and midguts, respectively, of HM (*w*AlbA*w*AlbB*w*Mel) mosquitoes, whereas *w*Pip and *w*AlbB were present in the highest and lowest levels, respectively, in HC ovaries. The densities of *w*AlbA and *w*Mel, but not *w*AlbB, were reduced from G6 to G18 in HM (*w*AlbA*w*AlbB*w*Mel) ovaries. These results indicate the existence of complicated interactions in term of both tissue tropism and CI expression when various *Wolbachia* strains co-exist in a host, providing important information to guide the design and establishment of transinfections in mosquito with optimal *Wolbachia* strains or their combination for disease control.

Our results indciate that competition for tissue colonization may occur between *Wolbachia* strains in the same supergroup. In the phylogeny of *Wolbachia*, both *w*Mel and *w*AlbA belong to supergroup A, whereas *w*AlbB and *w*Pip belong to supergroup B (Werren et al., 2008). With the introduction of *w*Mel into *Ae. albopictus* HOU mosquitoes carrying *w*AlbA and *w*AlbB, we observed that the density of *w*AlbA decreased by 7.8-fold in HM (*w*AlbA*w*AlbB*w*Mel) ovaries from G5 to G18, but *w*AlbB density remained stable. The level of *w*Mel infection also decreased from G5 to G18, but this decrease could have been caused by either the adaption of *w*Mel to a novel host background or competition from *w*AlbA, or both. Consistent with a previous report (Lu et al., 2012), the *w*AlbB density was higher than that of *w*AlbA in HOU ovaries. In the triply infected HC mosquitoes, generated by transfer of *w*Pip to HOU mosquitoes (Zheng et al., 2019), *w*AlbB was suppressed to a minimal level in the ovaries. Specifically, the density of *w*AlbB (5.6 × 10^–3^ *wsp*/*rps6*) was 7,934- and 5,226-fold lower than that of *w*Pip (44.6 *wsp*/*rps6*) or *w*AlbA (29.4 *wsp/rps6*), respectively. It is worth noting that this low number of *w*AlbB was still sufficient to induce CI, given that unidirectional CI has been observed in crosses of HC and GUA mosquitoes (Zheng et al., 2019). Thus, when *Wolbachia* is being introduced into an infected host, choosing a novel strain belonging to a supergroup different from that of the orginal infection may prove useful for avoiding competition. Caution should be used if the native strain provides an essential benefit to the host, since the novel strain will likely outcompete the native strain in the transfected line, based on our observations from the HM (*w*AlbA*w*AlbB*w*Mel) and HC lines.

Competition for CI induction can also occur among different strains within the same supergroup. Although a single *w*Mel infection is able to induce CI in both *Ae. aegypti* and *Ae. albopictus* (Walker et al., 2011; Blagrove et al., 2012), HM (*w*AlbA*w*AlbB*w*Mel) males did not induce CI when crossed with either GUA or HC females. After removal of *w*AlbA from the HM (*w*AlbA*w*AlbB*w*Mel) line, however, we observe a strong CI expression in crosses of HM2 (*w*AlbB*w*Mel) with either GUA, HC or HB. These results indicate that the ability of *w*Mel to modify the HM (*w*AlbA*w*AlbB*w*Mel) sperm may be blocked by the presence of *w*AlbA, instead of *w*Mel-modified sperm being rescued by *w*AlbA or *w*AlbB in HC or GUA mosquitoes. Consistent with our observations concerning HM2 (*w*AlbB*w*Mel) crosses, double infection of *w*AlbB and *w*Mel in transinfected *Ae. aegypti* was able to induce CI in the crosses with either non-infected, *w*AlbA-, *w*AlbB-, or *w*Mel-infected lines (Joubert et al., 2016). A similar effort to develop a triple infection (*w*AlbA, *w*AlbB, and *w*Mel) in *Ae. Albopictus* has been previously reported, but it resulted in very different outcomes: the triply infected line was self-incompatibility, its female was incompatible with wild-type male, and its male induced CI when crossed with wild-type females (Ant and Sinkins, 2018). It appears that the ability to rescue CI modification is compromised in their triply infected female, wherea the ability to induce CI is inhibited in our triply infected male. One possible explaination for the difference from our study is that the *w*AlbA density in the embryos of their triply infected line was inhibited to such an extent that it was impossible for *w*AlbA to rescue the CI modification in the males; in contrast, in our case the infection level of *w*AlbA was not significantly reduced in the HM (*w*AlbA*w*AlbB*w*Mel) ovaries when compared to wild-type. A similar experiment with different observation indicates complicated *Wolbachia*-host interactions when multiple strains coexsit and stresses the importance of repeating transinfection experiments with different genetic backgrounds of both donor and recipient strains as a way to obtain a useful combination of parameters.

Blocking by *w*AlbA of the *w*Mel-induced modification of sperm in the HM (*w*AlbA*w*AlbB*w*Mel) line suggests a potentional competition for host targets of CI factors between *w*Mel and *w*AlbA. Recent studies have suggested a “two-by-one” model underlying the CI mechanism in which *Wolbachia*-induced sperm modification is determined by two CI factors, *cifA* and *cifB*, whereas CI rescue is determined only by *cifA* (LePage et al., 2017; Shropshire et al., 2018; Beckmann et al., 2019; Chen et al., 2019). Further evidence has suggested that *cifB* targets nuclear protein import and protamine-histone exchange and that *cifA* rescues embryos by restricting the access of *cifB* to its targets (Beckmann et al., 2019). We hypothesize that the *cifB* genes of *w*Mel and *w*AlbA are very similar, so that they bind to the same sites that affect the host’s nuclear protein import and then are translocated to the nucleus, where their substrates for sperm modification reside. The affinity of native *w*AlbA for host targets may be higher than that of *w*Mel, thus preventing the *w*Mel from entering the nucleus to induce CI expression.

Very low rates of egg hatching were observed in the HM (*w*AlbA*w*AlbB*w*Mel) line before G6. Suprisingly, the outcross with wild-type HOU only increased egg hatch rates temporarily in some generations (e.g., G6, G17, and G23); in these cases, there was an immediate decline afterward, resulting in a fluctuation wave across 26 generations. When the HC line was initially established, low hatch rates were also observed for almost a year. The situation was different for the other transfected lines that we established, in that egg hatching quickly returned to a normal level after the outcrosses with wild-type for several consecutive generations (Xi et al., 2005a, b, 2006). It would presumably be more challenging for the host to establish a symbiotic relationship with a *Wolbachia* triple strain than with a single or double strain because of the overload of symbionts and the complicated interactions between various strains and the host. Interestingly, outcrosses of HM (*w*AlbA*w*AlbB*w*Mel) with another wild-type line, GUA, effectively recovered normal egg hatch rates, indicating that the GUA genetic background can facilitate the host’s adaptation to the novel triple infection. Because HM (*w*AlbA*w*AlbB*w*Mel) was derived from HOU, which had been maintained for a long time in the laboratory, outcrosses with HOU may not be able to introduce as much genetic heterogeneity to foster a novel symbiosis as can outcrosses with GUA, which was recently established from field samples (Zheng et al., 2019).

Here, we have demonstrated the successful extablishment of a transinfected *Ae. albopictus* HM (*w*AlbA*w*AlbB*w*Mel) line carrying a *Wolbachia* triple-strain infection. Unfortunately, the newly introduced *w*Mel strain failed to induce CI in this triply infected line, and our experimental evidence indicates that its ability to modify the sperm was blocked by the native strain, *w*AlbA. Further studies are needed to compare the CI determination factors associated with *w*Mel and *w*AlbA and to understand the molecular mechanism undergirding their potential competition in utilizing host targets for CI expression. The tissue tropism of the three *Wolbachia* strains in the HM (*w*AlbA*w*AlbB*w*Mel) line indicates their complicated interactions, with competition likely to happen between *Wolbachia* strains in the same supergroup. The differences in both CI expression and *Wolbachia* tissue tropism between the two triply transinfected lines HM (*w*AlbA*w*AlbB*w*Mel) and HC also indicate that caution is necessary when predicting the outcome of transinfected lines with multiple infections. These results provide important information to guide the future selection of *Wolbachia* strains for the development of transinfected lines in order to obtain the maximum pathogen-blocking efficiency, the lowest fitness cost, and ideal CI patterns.

## Data Availability Statement

The original contributions presented in the study are included in the article/Supplementary Material, further inquiries can be directed to the corresponding author.

## Author Contributions

XL and ZX contributed to conceptualization, methodology, validation, formal analysis, investigation, data curation, and original draft preparation. JL was responsible for CI crosses. GB performed embryo microinjection. ZX supervised the study. All authors contributed to the article and approved the submitted version.

## Conflict of Interest

JL and ZX were employed by Guangzhou Wolbaki Biotech Co., Ltd.

The remaining authors declare that the research was conducted in the absence of any commercial or financial relationships that could be construed as a potential conflict of interest.
